# Evaluation of commercially available small RNASeq library preparation kits using low input RNA

**DOI:** 10.1186/s12864-018-4726-6

**Published:** 2018-05-05

**Authors:** Ashish Yeri, Amanda Courtright, Kirsty Danielson, Elizabeth Hutchins, Eric Alsop, Elizabeth Carlson, Michael Hsieh, Olivia Ziegler, Avash Das, Ravi V. Shah, Joel Rozowsky, Saumya Das, Kendall Van Keuren-Jensen

**Affiliations:** 1 Cardiovascular Research Center, Massachusetts General Hospital, Harvard University, 185 Cambridge Street, Boston, MA 02114 USA; 2 Neurogenomics Division, Translational Genomics Research Institute, 445 N. 5th St, Phoenix, AZ 85004 USA; 3 Department of Molecular Biophysics and Biochemistry, Yale University, New Haven, CT 06520 USA

**Keywords:** Small RNASeq, Low RNA input, Small RNASeq, miRNA validation, NGS, qPCR

## Abstract

**Background:**

Evolving interest in comprehensively profiling the full range of small RNAs present in small tissue biopsies and in circulating biofluids, and how the profile differs with disease, has launched small RNA sequencing (RNASeq) into more frequent use. However, known biases associated with small RNASeq, compounded by low RNA inputs, have been both a significant concern and a hurdle to widespread adoption. As RNASeq is becoming a viable choice for the discovery of small RNAs in low input samples and more labs are employing it, there should be benchmark datasets to test and evaluate the performance of new sequencing protocols and operators. In a recent publication from the National Institute of Standards and Technology, Pine et al., 2018, the investigators used a commercially available set of three tissues and tested performance across labs and platforms.

**Results:**

In this paper, we further tested the performance of low RNA input in three commonly used and commercially available RNASeq library preparation kits; NEB Next, NEXTFlex, and TruSeq small RNA library preparation. We evaluated the performance of the kits at two different sites, using three different tissues (brain, liver, and placenta) with high (1 μg) and low RNA (10 ng) input from tissue samples, or 5.0, 3.0, 2.0, 1.0, 0.5, and 0.2 ml starting volumes of plasma. As there has been a lack of robust validation platforms for differentially expressed miRNAs, we also compared low input RNASeq data with their expression profiles on three different platforms (Abcam Fireplex, HTG EdgeSeq, and Qiagen miRNome).

**Conclusions:**

The concordance of RNASeq results on these three platforms was dependent on the RNA expression level; the higher the expression, the better the reproducibility. The results provide an extensive analysis of small RNASeq kit performance using low RNA input, and replication of these data on three downstream technologies.

**Electronic supplementary material:**

The online version of this article (10.1186/s12864-018-4726-6) contains supplementary material, which is available to authorized users.

## Background

Due to their stability, clinical relevance, and functional role in disease pathogenesis, small RNAs have the potential to be important reporters of dysregulated cellular processes across a range of diseases [1–3]. Their presence in biofluids has provided researchers with a new tool for monitoring health and disease, as these small RNAs, derived from intracellular sources throughout the body, make their way into circulation. RNA sequencing (RNASeq) is an attractive tool for small RNA discovery in both tissues and biofluids [4–6]. However, a general lack of reproducibility of miRNA results across laboratories and platforms [7–9] has been an area of concern when considering assays for small RNA quantitation at the outset of an experiment. The reasons for this lack of reproducibility can be attributed to several potential factors [10, 11]: 1) the nature of the samples themselves, notably differences in sample type and collection procedures, and heterogeneities in patient populations and patient stratification, 2) differences introduced at RNA or extracellular vesicle isolation steps, 3) variances introduced during data analysis, and 4) the inherent biases of the utilized technology; RNASeq, qPCR, microarrays and other assay platforms. In order to make informed choices at the initiation of experiments involving samples with low RNA input, we evaluated the performance of three commonly used RNASeq kits using commercially available standardized RNAs. In our assessment, we compared libraries prepared at two locations, using three different library preparation kits, at 1 μg or at 10 ng of RNA input (to mimic low input amounts found in biofluids). As the validation platforms for biofluids RNA have mostly focused on miRNAs, we then assessed the differentially-expressed miRNAs identified by RNASeq in downstream assays.

Biological diversity in the number and types of small RNAs present in a sample type may also play a role in the performance of the kits and downstream validation. It has become increasingly evident that tRNA fragments, YRNA fragments and other species of small RNAs are important cellular regulators and are abundant in biofluids [6, 12–14]. Therefore, to increase diversity, we used RNA isolated from three different tissues for comparison of low input RNA effects in the sequencing kits: brain, liver, and placenta, and one biofluid sample: plasma. Our results demonstrate that while small RNA expression differences between the three tissues, brain, liver and placenta are captured by all three small RNASeq kits tested (Illumina TruSeq, BiooScientific NEXTFlex, and New England Biolabs NEB Next), there are measurable differences between the kits in terms of RNA diversity. Moreover, while the inter-site variation was minimal for the 1 μg input samples, as the input amount was decreased to 10 ng, the effect of operator/site on the percentage of input reads mapping to RNA became more pronounced.

Validation of differentially expressed small RNAs, on other technologies, remains one of the most significant hurdles to its utility and clinical implementation. We assessed the performance of several platforms for validation of the RNASeq data, HTG EdgeSeq, Qiagen miRNome and Abcam FirePlex. Not surprisingly, we found that the performance of each platform correlated better when the expression level and fold change between the RNAs was larger.

As was suggested in a recent publication by Pine et al., 2018 [15], sequence and platform data from these samples can be used as a benchmark. These samples can be purchased from Thermo Fisher and used to test the overall performance of new sequencing protocols, small RNA quantitation platforms and lab-to-lab variability and operator proficiency. All of our data are available for comparison at SRA (BioProject: PRJNA402076).

## Results

**Fig. 1:**
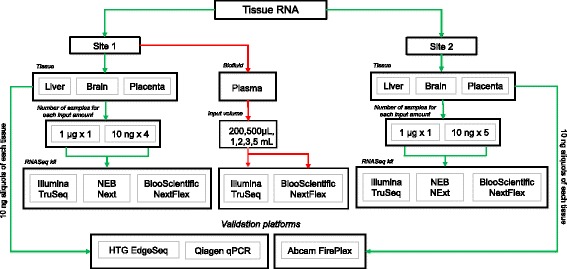
Schematic of study design. Tissue RNA from brain, liver and placenta were sequenced at two sites at two input amounts (1 μg and 10 ng) using three different RNA sequencing kits (Illumina TruSeq, NEB Next and BiooScientific NEXTFlex). RNA from plasma samples at 5 different input volumes (200 μL – 5 mL) were sequenced at Site 1 using only TruSeq and BiooScientific. The green arrow depicts the flow of one of the tissue samples – brain using NEB Next and the red arrows, the plasma samples. The RNASeq results from the tissue samples were then validated using three different platforms (qPCR, EdgeSeq performed by Site1 and Fireplex performed by Site2). For a full list of samples sequenced, please refer to Additional file 1: Table S1

**Fig. 2:**
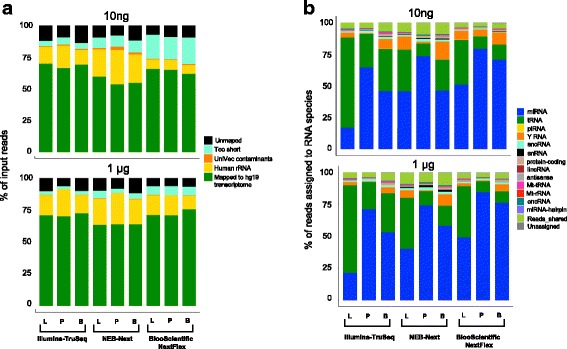
Distribution of total input reads and reads mapped to the transcriptome for liver (L), placenta (P) and brain (B). **a** Average percentage of input reads for each tissue and kit for the two input amounts (10 ng – top panel; 1 μg – bottom panel) aligned to the human transcriptome (hg19), UniVec contaminants, human rRNA, reads that were too short (< 15nts), and unaligned to the human transcriptome. **b** Average percentage of reads mapped to the human transcriptome to RNA biotypes for each tissue and kit for the two input amounts (10 ng – top panel; 1 μg – bottom panel): miRNA, tRNA, piRNA, YRNA, snoRNA, snRNA, protein-coding fragments, lincRNA (long intergenic non-coding RNA), antisense RNA, Mt_tRNA (mitochondrial tRNA), MT_rRNA (mitochondrial rRNA), oncRNA (other non coding RNA), miRNA hairpins, reads that are shared between multiple RNA biotypes and reads that are unassigned

We tested three commonly used small RNA sample preparation kits for next generation sequencing (TruSeq, NEB Next, and NEXTFlex), on three different tissues (brain, liver, and placenta), at two concentrations (1 μg and 100 fold less; 10 ng), and at two different sites (MGH and TGen). Figure 1 shows the schematic of the study design and the number of samples sequenced at each site for the two input amounts. Each tissue was sequenced once with each kit at the 1 μg RNA input. In order to test the variability of low RNA input for each of the kits, library preparation and sequencing was performed 8–10 separate times with 10 ng of input RNA. A summary of small RNASeq results is presented as an average across the two sites in Fig. 2a and b**.** Figure 2a displays the percentage of input reads that align to the human transcriptome (green), align to human rRNA (yellow), align to UniVec contaminants (orange), were too short after adaptor removal (< 15 nucleotides; turquoise), or did not align to the human transcriptome (black) for each tissue type and kit respectively (Fig. 2a).

The median percentage of reads aligned to the human transcriptome (after removing rRNA) for both 10 ng and 1 μg inputs were: Illumina TruSeq 74.12% (IQR: 70.7–79.63%), NEXTFlex 67.51% (IQR: 63.57–72.76%) and NEB Next 60.03% (IQR: 52.58–71.78%). Although there is no appreciable difference in the percentage of reads that were unmapped to the human transcriptome (~ 6.5%) and to UniVec contaminants (< 0.5%) across all three kits, the percentage of reads that mapped to human rRNA was significantly different (Illumina TruSeq – Median: 13.34%; IQR: 10.23–18.26%, NEXTFlex – Median: 9.5%; IQR: 7.23–11.21%, NEB Next – Median: 24.27%; IQR: 14.4–29.37%). The NEXTFlex kit had the highest percentage of reads that were too short (Median: 13.77%, IQR: 10.34–19.76%). A detailed sample list with the percentage of reads in each category can be found in Additional file 1: Table S1 and *p*-values calculated by Wilcoxon two-sided rank sum test with continuity correction for pairwise comparisons between the three kits is presented in Additional file 1**:** Table S2.

Reads mapped to the human transcriptome were further examined and the RNA categories/biotypes they mapped to are displayed in Fig. 2b for each tissue type and kit respectively. The most abundant RNA biotype in the placenta and brain samples is miRNA and in the liver samples, it is tRNA fragments (tRFs). Reads that mapped to tRFs predominantly arise from the 5 prime ends of tRNAs. Although all kits found a high representation of tRFs in the liver samples, Illumina TruSeq measured almost twice as many tRFs (TruSeq – Median: 69.48; IQR: 60.47–75.18, NEXTFlex – Median: 38.72; IQR: 35.56–44.7, NEB Next – Median: 32; IQR: 29.71–38.7). The NEXTFlex kit, with its 4 N adaptors, consistently had the highest number and diversity of reads assigned to miRNAs for all three tissues and input amounts [16, 17]. A Wilcoxon two-sided rank sum test between the three kits for the percentages assigned to the different RNA biotypes identified statistically significant differences between the tRNAs and miRNAs (Additional file 1: Table S4). The average for the miRNAs and tRFs account for 83, 81 and 78% of the reads mapped to the transcriptome for NEXTFlex, TruSeq and NEB Next respectively. The next most abundant RNA biotype is YRNAs that comprise, on average 6, 4 and 8% assigned to the three kits respectively. The light green bars at the top of Fig. 2b, depicted as ‘Reads_shared’, constitute reads that map to more than one biotype or position in the human transcriptome. These account for, on average, 5% of the reads for NEXTFlex, 7% for TruSeq and 8% for the NEB Next kit. A detailed sample list with the percentage of reads for each RNA biotype can be found in Additional file 1: Table S3 and *p*-values calculated by Wilcoxon two-sided rank sum test with continuity correction for pairwise comparisons between the three kits is presented in Additional file 1: Table S4.

### Effect of input amount

The effect of lowering the input amount of RNA 100 fold, from 1 μg to 10 ng, approximately doubled the percentage of reads that are categorized as too short (< 15 nts) for NEXTFlex (7 to 14%) and NEB Next (4 to 8%), while remained unchanged for TruSeq (2%) (Fig. 2a top panel versus bottom panel, Additional file 1: Table S5). The difference in the percentage of reads mapping to UniVec contaminants, although statistically significant for the NEXTFlex kit is still less than 0.5%. Median values of the percentage of reads assigned along with comparisons between the 1 μg and 10 ng input amounts are presented in Additional file 1: Table S5.

### Effect of site-to-site variation

While there are no statistically significant differences between the sites for the 1 μg input amount of RNA for all three kits (Additional file 1: Table S6), there are differences in the percentage of reads mapped to rRNA between the sites for the 10 ng samples NEXTFlex (9.6% vs 6.6%, Wilcoxon *P*-value < 0.01) and NEB Next (14.1% vs 29.7%, Wilcoxon *P* value < 0.001). The percentage of reads that were too short (<15nts) for TruSeq 10 ng input samples were also different between the two sites (2.1% vs 16.6%, Wilcoxon P value < 0.01). The percentage of input reads that map to the human transcriptome is significantly different between the two sites for the 10 ng samples for all three kits. Additional file 1: Table S7 shows the number of miRNAs that are detected at greater than 10 counts in at least 25% of the samples after normalization for sequencing depth by *DESeq2* [18] (See Methods) between the two sites for the two input amounts for all three kits. There is no statistically significant difference between the 1 μg and 10 ng samples for Illumina and NEB Next between the two sites. However, there is a significant difference in the number of miRNAs detected with the 10 ng input NEXTFlex samples between Site 1 (Median: 633; IQR: 580–654) and Site 2 (Median: 344; IQR: 319–352). Furthermore, a principal components analysis (PCA) shows that these samples cluster by Site and not by tissue type (Additional file 2: Figure S3); hence these 10 ng NEXTFlex samples from Site 2 were removed from further analyses. These data indicate that with smaller RNA inputs there are greater technical challenges from site to site, particularly related to the use of the NEXTFlex kit, and these differences are more pronounced for low abundance miRNAs,

### miRNA analysis

**Fig. 3:**
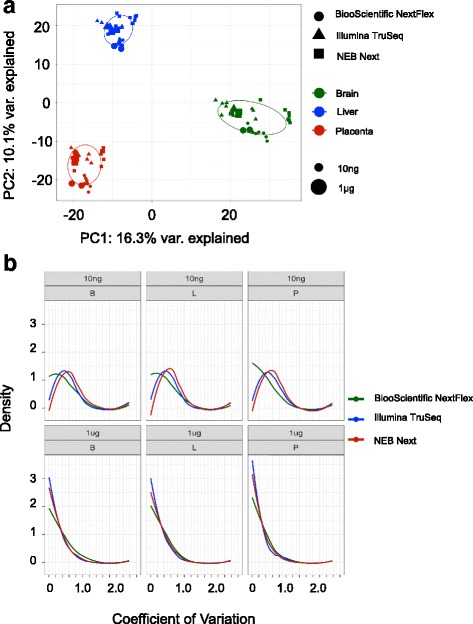
**a** Principal components analysis (PCA) plot of all samples based on their miRNA expression with the colors represent the three tissues (Brain – Green; Liver- Blue; Placenta - Red), the shape of the points representing the three kits (BiooScientific NEXTFlex – circle; Illumina TruSeq – triangle; NEB Next - square) and the size of the points representing the two input amounts. **b** Density plot of the coefficient of variation (CV) of all miRNAs for each kit and tissue for the two input amounts (10 ng – top panel; 1 μg – bottom panel)

When differences between the kits and the sites are included, the correlation between the 1 μg and 10 ng inputs of a tissue never drops below 0.88 (Additional file 1: Table S8). Biological differences between the tissues are captured reproducibly, no matter the sequencing kit or the RNA input. The PCA in Fig. 3a shows clear clustering of the three tissues for both the 10 ng and 1 μg input amounts across all three kits. Breaking down the data within each tissue cluster, there is some clustering of the samples by kit type. For example, the samples from brain tissue (green) are clustered together, well away from the other tissues, independent of kit or input amount. The large and small symbols, indicating input amount, cluster together by kit. Within this tissue cluster, the NEXTFlex samples (circles) all cluster at the bottom of the brain cluster, TruSeq (triangles) to the slight top left, and NEB Next (squares) to the top right. To evaluate the reproducibility of the kits, the coefficient of variation for each miRNA was computed per tissue for each input amount across the two sites and is displayed as a density plot in Fig. 3b**.** The peak density for the 1 μg samples for all three kits is near the x-axis, suggesting that the majority of the miRNAs have very low CVs. However, the peak density for the 10 ng samples have CVs between 0.4 and 0.6 for the NEB Next and Illumina TruSeq and begins closer to the x-axis for the NEXTFlex samples (CVs between 0 and 0.2) indicating that the lower input amount of RNA does increase the variability in the read counts assigned to miRNAs.

**Fig. 4:**
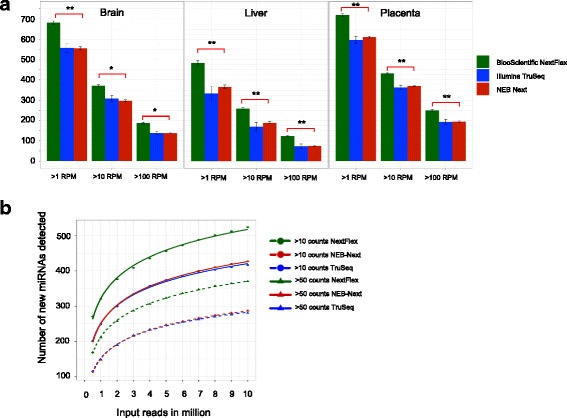
**a** Number of miRNAs detected at three expression thresholds: > 1 read per million mapped to the genome (RPM), > 10 RPM and > 100 RPM for each tissue and kit. The asterisks show significance for comparison between BiooScientific NEXTFlex and the other two kits. **b** Number of new miRNAs detected as a function of the number of input reads sequenced at two thresholds (> 10 read counts and > 50 read counts) for the three kits and tissues

**Fig. 5:**
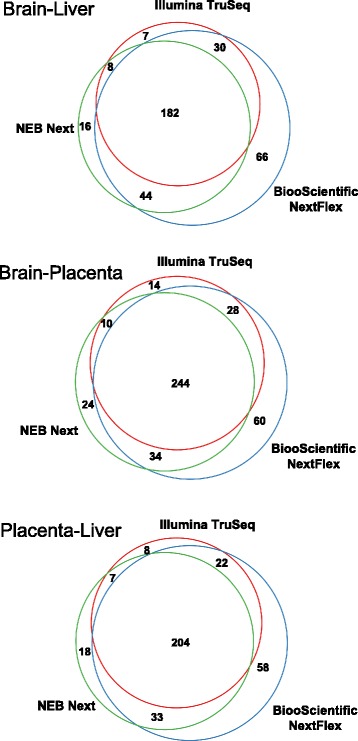
Venn diagrams representing the number of miRNAs that are differentially expressed between the three tissue comparisons – Brain versus Liver, Brain versus Placenta and Liver versus Placenta for all three kits

The number/diversity of miRNAs detected with the NEXTFlex kit is the highest. Figure 4a shows the number of miRNAs detected at three expression thresholds, 1, 10 and 100 RPM (reads per million mapped to the human transcriptome) for the three tissues and kits. In order to perform downstream analyses on the data, miRNAs should be robustly detectable and changes in expression should be reproducible across experiments. MiRNAs expressed above 50–100 RPM may be considered robustly expressed. As we are assessing the ability for all three kits to detect miRNA at low input amounts, we included data on lower levels of expression (1 and 10 RPM). There is no significant difference between the number of miRNAs detected between Illumina TruSeq and NEB Next for each of the three thresholds. Figure 4b elucidates the effect of sequencing depth on the detection rate of miRNAs. The rate of detecting new miRNAs at > 1 read count and > 10 read counts is depicted here as a function of sequencing depth ranging from 0.5 million to 10 million input reads for the three RNASeq kits with each additional million input reads bringing diminished return. The solid lines in Fig. 4b depict the number of new miRNAs detected as a logarithmic function of the number of input reads in millions. As we increase the number of input reads beyond 6 million, the rate of detection of new miRNAs greater than 50 counts drops below 10 miRNAs for each million reads added. NEXTFlex detects the highest number of new miRNAs at both > 1 and > 10 read counts, the difference between TruSeq and NEB Next is negligible. To assess the largest changes in individual miRNA detection across the kits, we list the top five miRNAs that had an expression of greater than 10 RPM in one kit and less then 5 RPM in the other two kits in Additional file 1: Table S9. Differential expression analysis for miRNAs that had robust expression (> 50 read counts in all tissues) was performed using *DESeq2* (See Methods). With the tissue specificity of the miRNAs exceeding the technical variability of the kits, expectedly, the majority of the statistically significant differentially expressed miRNAs (absolute fold change > 2 and adjusted *P* < 0.05) between pairwise combinations of the three tissues are in common between the three kits (Fig. 5). NEXTFlex had the highest numbers of differentially expressed miRNAs that were not detected in the other kits for all three pairwise tissue comparisons. This results from the fact that the NEXTFlex kit detected more miRNAs than the other two kits.

### Validation of differentially expressed miRNAs from RNASeq with Qiagen qPCR

The fold changes of the differentially expressed miRNAs (between different tissues) detected from RNASeq, for each pairwise tissue comparison, were compared to the fold changes obtained from three validation platforms: Qiagen miRNome miRNA PCR array, HTG EdgeSeq, and Abcam FirePlex. Only miRNAs that were robustly expressed in RNASeq (an average expression of > 50 normalized counts in at least 25% of the samples) with an absolute fold change > 2 and an adjusted *P*-value < 0.05 were used in the comparison. The three validation platforms each detect a subset of known miRNAs, and do not have the ability to distinguish between the canonical miRNA and their sequence or length isoforms (isomiRs).

**Fig. 6:**
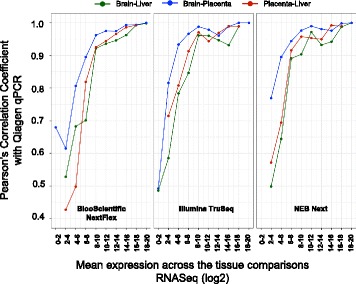
Pearson’s correlation coefficient computed from the log fold changes between RNASeq and Qiagen miRnome qPCR and plotted as a function of the average expression in RNASeq

Aliquots of RNA from each tissue were sent to Qiagen and measured on the Qiagen miRNome arrays, which allows for assessment of 1066 miRNAs. Table 1 lists the number of differentially expressed miRNAs identified through sequencing and the number of these miRNAs that were assayed by the Qiagen PCR array. MiRNAs are considered concordant if their fold change is in the same direction between RNASeq and qPCR and discordant if their fold change was in the opposite direction. There were 31, 3 and 29 miRNAs that were discordant between qPCR and all three sequencing kits for the Brain-Liver, Brain-Placenta and Placenta-Liver comparisons respectively. NEXTFlex, on average, had the highest number of miRNAs in common and concordant with the qPCR fold changes (NEXTFlex: 240, TruSeq: 192 and NEB Next: 222) and the highest number of discordant miRNAs with qPCR (NEXTFlex: 85, TruSeq: 73 and NEB Next: 75). NEB Next had the overall highest concordance with the PCR array results. Not surprisingly, the correlation of differentially expressed miRNAs between qPCR and RNASeq improves as the expression level of the miRNA and the fold change increases. This is illustrated in Fig. 6, the Pearson’s correlation coefficient between the log fold change in sequencing and qPCR is plotted as a function of the average read count expression in RNASeq. From the figure, differentially expressed miRNAs that have an average expression of greater than 2^8^ (256) read counts in sequencing correlate well (> = 0.9) with fold changes from qPCR. These data provide a guide for the expression threshold of miRNAs discovered via sequencing - for validation on high throughput qPCR platforms such as the Qiagen miRNome arrays.

**Table 1:** Number of miRNAs differentially expressed in RNA sequencing validated by Qiagen miRnome high throughput qPCR^a^

Kit	Tissue Comparison	Differentially Expressed miRNAs (RNASeq)^a^	Assayed by qPCR	Concordant – in same direction as RNASeq (%)	Discordant – in opposite direction as RNASeq (%)
BiooScientific	B_vs_L	322	285	209 (73.33%)	76 (26.67%)
BiooScientific	B_vs_P	366	305	299 (98.03%)	6 (1.97%)
BiooScientific	P_vs_L	317	269	214 (79.55%)	55 (20.45%)
Illumina-TruSeq	B_vs_L	227	207	164 (79.23%)	43 (20.77%)
Illumina-TruSeq	B_vs_P	295	254	250 (98.43%)	4 (1.57%)
Illumina-TruSeq	P_vs_L	241	206	162 (78.64%)	44 (21.36%)
NEB	B_vs_L	250	226	186 (82.3%)	40 (17.7%)
NEB	B_vs_P	312	269	262 (97.4%)	7 (2.6%)
NEB	P_vs_L	262	219	179 (81.74%)	40 (18.26%)

^a^ The differentially expressed miRNAs in RNA sequencing have an average expression (calculated across both tissues denoted in the “Tissue comparison” column) ≥ 50 counts and is expressed in at least 25% of the total number of replicates for each pairwise comparison. The percentage of concordant and discordant miRNAs are calculated based on the number of miRNAs assayed by qPCR

### Validation of differentially expressed miRNAs from RNASeq with HTG EdgeSeq

**Fig. 7:**
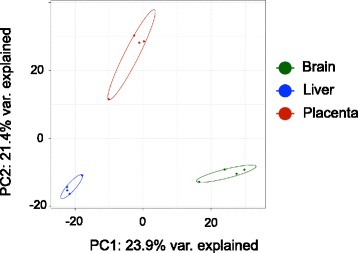
PCA plot of the three tissues based on their miRNA expression assayed by EdgeSeq

Aliquots of RNA from each tissue sample was sent to HTG for analysis on their HTG EdgeSeq platform. The HTG EdgeSeq library that we used for comparison to conventional sequencing surveyed 2085 human miRNA. The three tissues were sequenced with HTG EdgeSeq in triplicate with an input amount of 20 ng and one set was sequenced with an input amount of 10 ng. The number of miRNAs detected by HTG EdgeSeq at greater than 10 RPM was 946 ± 51 (mean ± standard error) and 325 ± 20 at greater than 100 RPM. There were no statistically detectable differences in the number of miRNAs detected between the 20 ng and the 10 ng inputs, therefore, all replicates were included as one analysis. PCA (Fig. 7) demonstrates the tissue specificity of the miRNAs. Differential expression analysis for miRNAs that had non-zero expression in at least 25% of the samples was performed using *DESeq2* (See Methods). Table 2 lists for each of the three pairwise combinations of the tissues in each kit, the number of differentially expressed miRNAs in RNASeq, the number of miRNAs assayed by HTG EdgeSeq, the number of miRNAs that were concordant and discordant with RNASeq and the number of miRNAs that were concordant and significantly differentially expressed (absolute fold change > 2 and an adjusted *P* < 0.05) in both HTG EdgeSeq and RNASeq. On average, about 85% of the differentially expressed miRNAs in RNASeq are detected by HTG EdgeSeq with greater than 95% of the miRNAs being concordant with RNASeq. NEXTFlex, on average has the highest number of miRNAs that were concordant with HTG EdgeSeq (NEXTFlex: 215, TruSeq: 177 and NEB: 187). Of the miRNAs that were not significant in HTG EdgeSeq, but were significant in RNASeq, 82% were in agreement with RNASeq regarding the directionality of the fold change. Approximately 30% of the miRNAs significant by RNASeq, but not significant by HTG EdgeSeq, had an average expression < 100 counts in RNASeq.

**Table 2:** Number of miRNAs differentially expressed in RNA sequencing validated by EdgeSeq^a^

Kit	Tissue Comparison	Differentially Expressed miRNAs (RNASeq)^a^	Assayed by EdgeSeq	Concordant – in same direction as RNASeq (%)	Discordant – in opposite direction as RNASeq (%)	Concordant and Significant (%)
BiooScientific	B_vs_L	322	292 (90.68%)	267 (91.44%)	25 (8.56%)	210 (71.92%)
BiooScientific	B_vs_P	366	305 (83.33%)	294 (96.39%)	11 (3.61%)	243 (79.67%)
BiooScientific	P_vs_L	317	264 (83.28%)	252 (95.45%)	12 (4.55%)	193 (73.11%)
Illumina-TruSeq	B_vs_L	227	204 (89.87%)	197 (96.57%)	7 (3.43%)	173 (84.8%)
Illumina-TruSeq	B_vs_P	295	243 (82.37%)	235 (96.71%)	8 (3.29%)	202 (83.13%)
Illumina-TruSeq	P_vs_L	241	195 (80.91%)	184 (94.36%)	11 (5.64%)	156 (80%)
NEB	B_vs_L	250	224 (89.6%)	218 (97.32%)	6 (2.68%)	179 (79.91%)
NEB	B_vs_P	312	258 (82.69%)	251 (97.29%)	7 (2.71%)	213 (82.56%)
NEB	P_vs_L	262	213 (81.3%)	203 (95.31%)	10 (4.69%)	168 (78.87%)

^a^ The differentially expressed miRNAs in RNA sequencing have an average expression (calculated across both tissues denoted in the “Tissue comparison” column) ≥ 50 counts and is expressed in at least 25% of the total number of replicates for each pairwise comparison. The percentage of concordant and discordant miRNAs are calculated based on the number of miRNAs assayed by EdgeSeq. Significant miRNAs in EdgeSeq: abs(log_2_FC) > 1 and p_adj_ < 0.05

### Validation of differentially expressed miRNAs from RNASeq with Fireplex

An aliquot of RNA from each tissue was sent to Abcam for testing on their FirePlex, hybridization and flow sorting platform. A total of 131 miRNA probes were included on a custom made FirePlex panel for the three tissues and the samples were run in triplicates. A full list of the probes and the number of tissues in which they were detected is presented in Additional file 1: Table S10. Nineteen miRNA detected by sequencing were below the limit of detection for FirePlex. 32% (6/19) of the undetected FirePlex miRNA probes had an average expression of less than 100 counts in RNASeq. A two-tailed t-test was used to carry out differential expression analysis for pairwise comparisons of the tissues and a nominal *P* < 0.05 was used to determine significance. On average, ~ 70% of the miRNAs that were detected by FirePlex were concordant with RNASeq in both directionality and statistical significance. Of the miRNAs that were measured to be significantly differentially expressed by sequencing, but not by FirePlex (*p*-value> 0.05), a significant proportion of them (87%) were in agreement with RNASeq regarding the directionality of the fold change. Table 3 summarizes the comparison between RNASeq and FirePlex.

**Table 3:** Number of miRNAs differentially expressed in RNA sequencing validated by FirePlex^a^

Kit	Tissue Comparison	Differentially Expressed miRNAs (RNASeq)^a^	Assayed by FirePlex	Concordant – in same direction as RNASeq (%)	Discordant – in opposite direction as RNASeq (%)	Concordant and Significant (%)
BiooScientific	B_vs_L	322	47	39 (82.98%)	8 (17.02%)	28 (59.57%)
BiooScientific	B_vs_P	366	54	52 (96.3%)	2 (3.7%)	35 (64.81%)
BiooScientific	P_vs_L	317	42	41 (97.62%)	1 (2.38%)	32 (76.19%)
Illumina-TruSeq	B_vs_L	227	43	39 (90.7%)	4 (9.3%)	29 (67.44%)
Illumina-TruSeq	B_vs_P	295	53	50 (94.34%)	3 (5.66%)	35 (66.04%)
Illumina-TruSeq	P_vs_L	241	38	38 (100%)	0 (0%)	31 (81.58%)
NEB	B_vs_L	250	45	39 (86.67%)	6 (13.33%)	29 (64.44%)
NEB	B_vs_P	312	51	50 (98.04%)	1 (1.96%)	34 (66.67%)
NEB	P_vs_L	262	39	39 (100%)	0 (0%)	31 (79.49%)

The differentially expressed miRNAs in RNA sequencing have an average expression (calculated across both tissues denoted in the “Tissue comparison” column) ≥ 50 counts and is expressed in at least 25% of the total number of replicates for each pairwise comparison. The percentage of concordant and discordant miRNAs are calculated based on the number of miRNAs assayed by FirePlex. Significant miRNAs in FirePlex: *p* < 0.05

### Comparison of RNASeq kits for extracellular plasma RNA

**Fig. 8:**
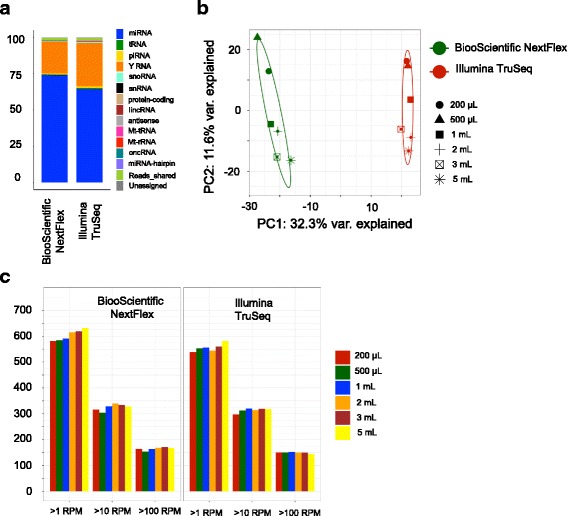
**a** Average percentage of reads mapped to the human transcriptome to RNA biotypes for Illumina TruSeq and BiooScientific NEXTFlex for the plasma samples. The percentages presented here are averaged over the 6 different volumes for each kit respectively. **b** PCA plot of the plasma samples show clustering of the samples by kit-type and not by input volume. **c** Number of miRNAs detected at three expression thresholds: > 1 read per million mapped to the genome (RPM), > 10 RPM and > 100 RPM for all the plasma samples for Illumina TruSeq and BiooScientific NEXTFlex

We next sought to compare low input RNASeq from a different sample type; plasma. We chose two kits; TruSeq (results for TruSeq and NEB Next were very similar for all of the tissue comparisons) and the NEXTFlex kit (because it was the most different from the other two kits). We examined the effect of plasma input volume on sequencing results. Total extracellular RNA was isolated from five different volumes of a large pooled plasma sample, from 200 μL to 5 mL. Figure 8a is a stacked bar plot that shows the average percentage of reads that map to different RNA biotypes for NEXTFlex and TruSeq. The NEXTFlex kit has a higher percentage of reads that align to miRNAs (NEXTFlex: 74%; TruSeq: 64%). The remaining reads map to YRNA fragments and together, the miRNAs and YRNA fragments make up ~ 95% of the sequencing from these plasma samples in both kits. The samples segregate by kit type, and not by their input volumes (Fig. 8b), revealing that the difference in the kits is greater than the difference in input volume. The low input volumes (200 μL, 500 μL) and the high input volumes (2, 3 and 5 mL) do cluster differently. As with the tissue samples, the number of miRNAs detected for NEXTFlex at three different thresholds, > 1, 10 and 100 RPM, is greater than TruSeq (Fig. 8c).

## Discussion

Investigators interested in small RNAs have a number of choices regarding which of the protocols and kits for RNA isolation, library sample preparation, and downstream validation platforms would be most suitable for their experiments. The findings of the miRNA Quality Control Study described significant differences among platforms for miRNA detection [9]. We were further concerned about the potential exacerbation of small RNASeq bias and lack of concordance when using samples with low RNA inputs [17, 19]. Therefore, we wanted to characterize sources of variation in small RNASeq from low input amounts of RNA, and correlate these results with downstream validation platforms. These studies could help identify strategies that could increase the rigor and reproducibility of data across projects and laboratories.

We assessed the data quality from small RNASeq when reducing RNA input by 100× from 1 μg to 10 ng, and the correlation of the sequencing results from these 10 ng samples with downstream validation platforms. We used three different tissues, brain, liver and placenta to examine differences in RNA expression across three sequencing kits (NEB Next, NEXTFlex, and TruSeq). We then took the differential expression patterns between tissues and assayed them on platforms that used qPCR (Qiagen miRNome assay) or miRNA hybridization strategies with different readouts, sequencing with HTG EdgeSeq and flow sort using Abcam FirePlex. To enable comparisons across the kits, all data analyses were performed identically on all samples.

While each of the kits have inherent sequencing biases for a subset of detected RNAs (Additional file 1: Table S9), these kit specific differences were minor compared with biological differences in each tissue. It is worth noting that the TruSeq kit detected far more tRNA fragments than the other kits. It is unclear what the true proportion of tRNA fragments are in the liver samples and this should be further tested. While we were able to discern minor differences for each kit and its performance, reassuringly the majority of miRNA data correlated well across kits for each tissue and input amount. Minor differences between the kits were identified and should be considered when choosing a kit for a project. More miRNA was detected in samples sequenced with the NEXTFlex kit at both input concentrations. However, this kit also showed larger site-to-site differences, and a higher number of reads that were too short, especially when the input RNA amount was 10 ng.

Expression thresholds are an important consideration for miRNA studies. For studies profiling and discovering small RNA biotypes as potential readouts of health and disease, it may be useful to identify and report RNAs expressed at low levels across a variety of tissues and biofluids – especially while the field is in the early phases of discovery. It is currently unknown what threshold is appropriate for biological relevance or diagnostic purposes for small RNA. For example, RNAs discovered via sequencing that have low numbers of counts, may still be robustly detected when using a targeted approach. Notably, however, higher levels of expression will be more accurately detected across sites, studies, and platforms. We identified miRNAs differentially expressed between two tissues with > 50 read counts. We then assessed these miRNA changes on other platforms. Not surprisingly, miRNA that have low levels of expression and are identified as differentially expressed between two tissues by sequencing, are harder to validate with one another and on other platforms (Fig. 6), compared to miRNA that are highly expressed with large fold changes. Using 50 read counts as the cutoff threshold, approximately 56% of all miRNAs found to be differentially expressed were common across all three kits.

Biological differences were preserved and validated across all platforms. For example, brain compared with placenta had the largest number of differentially expressed miRNA detected by RNASeq. The differentially expressed miRNAs in these two tissues were also consistently detected with the highest concordance in each platform (Tables 1, 2 and 3). We were generous with the term validation; downstream platforms needed to measure changes in expression in the same direction as RNASeq. If any of these downstream assays were to be turned into validation platforms or diagnostic tools, they would need to be optimized and further assessed for their ability to detect similar significant fold changes. The HTG EdgeSeq results were closest to the RNASeq results with greater than 95% concordance (Table 2), potentially because the readout of the platform itself is sequencing.

The Qiagen qPCR high-throughput assay performs reasonably well as a validation protocol, with on average, ~ 85% of the miRNAs in agreement with RNASeq. HTG EdgeSeq is based on next generation sequencing technologies; the HTG EdgeSeq library preparation enriches for miRNAs by probe-based capture and has on average ~ 80% concordance with RNASeq. The majority (~ 82%) of the remaining miRNAs that were not statistically significant in HTG EdgeSeq have fold changes in the same direction as RNASeq. FirePlex too utilizes a probe-based miRNA enrichment strategy with no separate RNA isolation step. There is a reasonably high level of concordance with RNASeq (~ 70%), with the majority of the fold changes of miRNAs that did not reach significance, in the same direction as in RNASeq. However, it is important to keep in mind that all three technologies discussed here do not have the ability to distinguish sequence or length variations or isoforms of miRNAs (isomiRs) from the canonical mature miRNA.

It appears that as the average expression of the miRNAs in RNASeq increases beyond 250–300 RPM, there is a good correlation with both Qiagen qPCR and HTG EdgeSeq as validation platforms. This is especially useful to be aware of when working with extracellular RNA from biofluid samples where the miRNA profiles are dominated by a few miRNAs with orders of magnitude more expression compared to the remaining miRNAs.

We also assessed small RNASeq with plasma samples at different volumes. For research purposes, plasma sample volumes are typically limited so that many different assays and analyses can be performed using the same sample. If the RNAs related to a disease were identified and present in plasma for use as a diagnostic, acquiring larger volumes would not be a problem. However, we tested a range of volumes for RNA input consistent with what is used for research and discovery, from 200 μL to 5 mL. We found that the data across all volumes correlated very well. The smallest volumes tended to cluster slightly away from the larger volumes. However, it should be noted that these findings were from freshly isolated good quality plasma; whether these findings hold up for archival samples of variable age was not explored.

There are a growing number of studies that have identified small RNAs as pivotal to normal cellular function and development of disease [20–22], yet reproducibility of data across laboratories has been challenging [3, 23]. In addition, there has become an enthusiastic interest in extracellular RNAs and what types of small RNAs are in circulation [4, 6, 12–14]. Many of these areas of investigation are limited by the RNA available, necessitating a careful examination of how well kits and platforms perform with low RNA input. We need to explore and identify the boundaries to what is robust and reproducible in low input experiments. One area of potential challenge to data reproducibility is in the types of discovery and validation platforms used. We tested the effects of low RNA input on the robustness of data generated from RNASeq. Our major finding was a significant increase in the number of miRNAs detected using the NEXTFlex kit, that would have an impact on downstream analysis and validation. While our paper was under review, another paper from Dard-Dascot et al., 2018 [24], found similar results when comparing five different sequencing kits. They also concluded that the NEXTFlex kit provided a higher diversity of miRNA.

All of these data are available and can be combined with the data from Pine et al., 2018 [15] using the same samples. Performance of new sequencing protocols and platforms can be compared with this data to assess performance.

## Conclusions

The results indicate 4 important findings: 1) We recommend using the BiooScientific NEXTFlex kit, as it detects the largest number of miRNAs, owing to its 4 N random adaptor sequence that ameliorates ligation bias. However, there are challenges to implementing this kit with low input RNA. When using this kit, both sites found an increased amount of reads that were too short to be used in downstream analysis, the kit required more experience and familiarity before technicians were able to use it consistently. 2) Reassuringly, the biological difference between tissues for miRNAs exceed that of the difference in input RNA or between the three small RNA sequencing kits tested here. 3) The validation of findings from RNA sequencing experiments by other technologies such as qPCR, EdgeSeq and FirePlex is principally dependent on the expression levels in RNA sequencing; the higher the expression and fold change of the RNA, the more likely it will be validated in other technologies. 4) Small RNA sequencing from freshly extracted plasma samples can be carried out efficiently with input volumes as low as 200 μL with over 300 miRNAs detected at > 10 read counts. These data demonstrate for the first time, a multi-site quantitative analysis of miRNA discovery via RNA sequencing for low input RNA amounts and subsequent validation on three distinct platforms.

## Methods

### Samples

The three total human RNA sources distributed were obtained from Ambion (Thermo Fisher Scientific): Human Brain Reference RNA (Cat. No. AM6050), Human Liver Total RNA (Cat. No. AM7960) and Human Placenta Total RNA (Cat. No. AM7950) Two concentrations of the total RNA were used in each library preparation. RNA was quantified in triplicate using Quant-iT Ribogreen RNA Assay kit, Low-Range protocol (R11490; ThermoFisher). We used 1 μg, which is the staring input recommended by each sequencing kit, and 10 ng. A large pool of plasma (30 mL) was used for comparison of kits and input volumes. Whole blood was collected from normal healthy volunteers in EDTA tubes, spun at 2500 rpm to separate plasma. Plasma was pooled, and 1 mL aliquots placed in tubes and stored at − 80 °C. The appropriate number of tubes were combined and isolated for each comparison. For example, for the 5 mL input volume, 5 aliquots were selected and the RNA isolated (using miRVana Paris, ThermoFisher AM1556, with modifications [25] and sample preparation for sequencing performed. The samples were collected in accordance with established Institutional Review Board protocols (WIRB #20142635).

### Sequencing

#### Sequencing was performed at two sites: MGH and TGen

##### Illumina TruSeq small RNA library preparation

Small RNA libraries were generated using Illumina TruSeq Small RNA Sample kit (RS-200-0048; Illumina). The reagents were utilized with the following modifications. The 3′ adapter, 5′ adapter, Stop Solution, RNase Inhibitor and RT primer were diluted by 50% with water [25]. During PCR amplification, the Index primer and RNA PCR primer volumes were reduced by 50% and the volume replaced with water. PCR amplification was performed based on the upper and lower recommendation of the kits. A total of 16 cycles for the 10 ng starting material and 11 cycles for the 1 μg starting material. The libraries were then run in a 6% TBE Gel for 55 min at 140 V and the between 140 to 160 bp were excised from the gels. These gel pieces were fractured into smaller pieces and allowed to incubation on a rotator overnight in water. Then an ethanol precipitation was performed to precipitate the RNA and the resulting pellet of RNA was resuspended in 11ul of ultra pure water.

##### BiooScientific NEXTFlex small RNA library preparation

Small RNA libraries were generated using NEXTFlex Small RNA Library Prep Kit v2 (Cat #5132–03). The manufacturer’s instructions where followed through PCR amplification. The optional stop point was used post the RT reaction prior to clean up and PCR amplification. In regards to PCR amplification the 1 μg samples received 12 cycles and the 10 ng sample received 18 cycles. Following PCR amplification, the libraries where then run in a 6% TBE Gel for 30 min at 200 V and the between 150 to 170 bp were excised from the gels. At this point there was a divergence from the manufactures instruction, an ethanol precipitation was used in place of the recommenced bead purification. The resulting pellet of RNA was resuspended in 11 μl of ultra pure water.

##### NEB small RNA library preparation

Small RNA libraries were generated using NEB Next Small RNA Library Prep Set for Illumina (NEB #E7330S). The manufacturer’s instructions where followed through with the PCR amplification. The PCR amplification was done under the parameters of 1 μg starting input received 12 cycles and the 10 ng input received 15 cycles. Following PCR amplification, the libraries where then run in a 6% TBE Gel for 60 min at 120 V and the between 140 to 160 bp were excised from the gels using a razor blade. Diverging from the manufacture protocol, gel pieces were fractured into smaller pieces and allowed to incubation on a rotator overnight in water. Then an ethanol precipitation was performed to precipitate the RNA and the resulting pellet of RNA was resuspended in 11ul of ultra pure water.

##### Small RNA library QC and pooling

Samples were quantified with the Agilent High Sensitivity DNA Kit (5067–4626; Agilent). The peak for the sample was integrated from 120 bp to 160 bp to get the pMolarity of the product that will cluster on the sequencer. Samples with similar pMolarity were grouped on the same lane and pooled together to create a total of 8 unique pools containing 15 samples with different barcodes. These pools were then quantified with the Agilent High Sensitivity DNA Kit to get the final pMolarity for the pools. The pools were then denatured and clustered on a single read Illumina V3 flowcell (GD-401-3001; Illumina). Site 1 flowcells ran on the Illumina HiSeq sequencing platform (HiSeq 2000/2500; Illumina) for 50 cycles for with a 7 cycle indexing read. The samples from Site 2 were sequenced on an Illumina NextSeq 500 for single read 76 cycles. Site 2 sequenced to a read length of 76 nts, whereas Site 1 samples were sequenced to <=50 nts. To ensure a fair comparison between the RNA fragments detected by the two sites, only reads that were in the range of 15–50 nucleotides were considered. Density plots of the unrestricted read length distributions from both sites are presented in Additional file 2: Figure S1**.** The difference in the percentages of reads assigned to the various RNA biotypes when the read length is restricted to <=50 nts for the Site 2 samples is shown in Additional file 2: Figure S2**.**

### HTG EdgeSeq

Samples were sent to HTG for sequencing. The HTG EdgeSeq miRNA Whole Transcriptome Assay was used to run the NIST RNA samples as described previously [26], with the following exception, the nuclease protection assay was run at 41 °C rather than 50 °C. Briefly, Lysis Buffer (HTG) was added to each of the 18 NIST purified RNA samples, and either 10 ng (both versions) or 20 ng (version 1 only) per sample was used in the assay. Each sample was run in triplicate. Following nuclease protection, each sample was tagged individually with molecular barcodes; tagged samples were pooled and sequenced on an Illumina MiSeq sequencer. Fastq files from the individual replicates were processed and the expression data reported as raw counts by the HTG EdgeSeq parser software. Data from one replicate of one sample did not pass QC metrics and were excluded from the analysis.

### Abcam FirePlex

Samples were processed using the FirePlex miRNA Assay (Abcam) as per the published protocol [27].

In brief, for each tissue sample, 1 ng total RNA was loaded. The plate was incubated at 37 °C for 60 min with shaking. After rinsing twice with 1X Rinse A, 75 μL of 1X Labeling Buffer was added to each well. The plate was incubated at room temperature for 60 min with shaking. After two rinses with 1X Rinse B followed by one rinse with 1X Rinse A, a catch plate was added to the vacuum manifold and the filter plate put under constant vacuum. 65 μL of 95 °C RNAse-free water was added twice to each well to elute the ligated sample. 30 μL of this meltoff was added to a clean PCR plate and mixed with 20 μL PCR master mix. The mixture underwent 32 cycles of PCR amplification. Next 60 μL of Hybe Buffer was added back to each well of the original particles followed by 20 μL of the PCR product, and the plate was incubated at 37 °C for 30 min with shaking. After rinsing twice with 1X Rinse B followed by one rinse with 1X Rinse A, 75 μL of 1X Reporting Buffer was added to each well and the plate incubated at room temperature for 15 min with shaking. After rinsing twice with 1X Rinse A, 175 μL of Run Buffer was added to each well. The samples were then scanned on an EMD Millipore Guava 6HT flow cytometer. The flow cytometer output was analyzed with the FirePlex Analysis Workbench software.

### Qiagen miRNome PCR Array

Samples were evaluated with real-time PCR using the miScript PCR System (Qiagen, Venlo, Netherlands). Briefly, 10 ng of each sample was reverse transcribed and then preamplified (13 cycles) using the miScript Single Cell qPCR Kit, according to the manufacturer’s protocol. Following preamplification, the 25 μl amplification product was diluted to 127 μl using nuclease-free water. From there, 110 μl of the diluted product was diluted to a 330 μl final volume using nuclease-free water. Real-time PCR was then performed using the human miRNome (QIAGEN catalog number MIHS-3216Z). 100 μl of diluted product was applied to each of the three, 384-well plates associated with the miRNome. Real-time PCR was performed on a ThermoFisher instrument using the recommended miScript cycling parameters.

### RNASeq data analysis

The raw sequence image files from the *Illumina HiSeq 2500* or *Illumina MiSeq* in the form of *.bcl* are converted to the *fastq* format using *bcltofastq v1.8.4* and checked for quality to ensure the quality scores do not deteriorate drastically at the read ends. The adapters from the 3′ end are clipped using *cutadapt* v.1.10 [28] (http://cutadapt.readthedocs.io/en/stable/guide.html). Reads shorter than 15 nts are discarded and after adapter trimming, the 3′ bases below a quality score of 30 are trimmed as well.

Reads that arise from human rRNA and contamination from library preparation protocols are removed before they are mapped to the human genome. The reads are first mapped to a library of UniVec contaminants, a collection of common vector, adapter, linker and PCR primer sequences collated by the NCBI. They are then mapped to human rRNA sequences obtained from NCBI The reads are mapped to the rRNA and UniVec sequences using *Bowtie2* [29] and those that map are removed from the analysis. The alignment to the human genome and transcriptome takes place in two stages. First, the rRNA and UniVec free reads are mapped to the human genome (hg19) using *STAR* [30]*.* The reads that map to the genome are then mapped to the human transcriptome. Also, the reads that are not mapped to the human genome are mapped to the human transcriptome, The library for the human transcriptome is built by concatenating miRNAs and hairpins from miRBase 21 [31], tRNAs from gtRNAdb [32], piRNAs from piRBase v1.0 [33], protein-coding, non-coding and and other RNA sequences from *ENSEMBL 75*. The *STAR* alignment is performed end to end with a single mismatch allowed while mapping and each read is allowed to multimap to at most 40 RNA annotations. Here, there is no mismatch allowed and each read is allowed to multimap to at most 40 RNA annotations. Additional file 1: Table S11 provides the list of libraries used and their versions.

### Differential expression analysis

For both RNASeq and EdgeSeq, differential expression was conducted for all miRNAs that had expression in at least 25% of the samples across the two groups that were compared with the DESeq2 [29] package in R. The raw read counts for the samples were normalized using the median ratio method (default in DESeq2). For the RNASeq analysis, miRNAs were deemed to be statistically differentially expressed if they had an expression of greater than 50 counts in at least 25% of the samples at an absolute fold change > 2 and an adjusted *P* < 0.05 (Benjamini Hochberg). For the EdgeSeq analysis, miRNAs were deemed to be statistically differentially expressed if they had an absolute fold change > 2 and an adjusted *P* < 0.05 (Benjamini Hochberg). For the FirePlex analysis, the data was log-transformed after adding a pseudo value of 1 and two-tailed t-tests were used to determine significance at a nominal *P* < 0.05.

## Additional files

Additional file 1: **Table S1.** Percentage of input reads aligned to the human transcriptome, human rRNA, UniVec contaminant sequences and discarded because they are too short (< 15 nts) and unmapped to the human transcriptome. **Table S2.** Median (Inter-quartile range) of percentage of input reads aligned to the human transcriptome, human rRNA, UniVec contaminant sequences. **Table S3.** Percentage of reads aligned to the human transcriptome to each RNA biotype for all samples. **Table S4.** Median (Inter-quartile range) of percentage of input reads aligned to different RNA biotypes between the three sequencing kits. **Table S5.** Median (IQR) of percentage of input reads aligned and comparison of input amount of RNA. **Table S6.** Median (IQR) of percentage of input reads aligned and comparison between the two sites for the two input amounts of RNA. **Table S7.** Median (IQR) of number of miRNAs greater than 10 counts detected in at least 25% of the samples between the two sites for the two input amounts of RNA. **Table S8.** Pearson’s and Spearman’s correlation coefficient by tissue, kit and input amount. **Table S9.** Kit specific miRNAs found in each tissue for each kit. The top 5 miRNAs for each tissue that have expression greater than 10 RPM in one kit, but less than 5 RPM in the other two are presented for each tissue and kit. **Table S10.** miRNAs included on the custom made FirePlex Panel. The columns denote the number of samples that had above detection-limit expression in each tissue. **Table S11.** Database of RNA biotypes used. (XLSX 89 kb)

Additional file 2: **Figure S1.** Density plot of read lengths for all three kits and tissues respectively by site. Site2 sequenced to a length of 76 nts, whereas all of Site1 samples were sequenced to <=50 nts. **Figure S2.** Comparison of percentage of reads assigned to the various RNA biotypes for read length restricted to less than 50 nts versus read length = 76 nts. Site2 sequenced to a length of 76 nts. **Figure S3.** PCA plot showing that the BiooScientific NEXTFlex samples from Site2 cluster by themselves indicating a batch effect. Also, the figure on the right shows the number of miRNAs detected > 10 counts for the two input amounts 10 ng and 1 μg by Site for the BiooScientific NEXTFlex samples. (PDF 5418 kb)

## Abbreviations

isomiRs: isoforms of miRNAs
miRNA: micro RNA
PCA: Principal Components Analysis
qPCR: quantitative polymerase chain reaction
RNASeq: Ribonucleic Acid Sequencing
RPM: Reads per million
rRNA: ribosomal RNA
tRFs: transfer RNA fragments
