# Supplementary material for: Evaluation of commercially available small RNASeq library preparation kits using low input RNA
Source: BMC Genomics. 2018 May 5;19:331. doi: 10.1186/s12864-018-4726-6 (PMC5936030; doi:10.1186/s12864-018-4726-6)

Figure S1 Density plot of read lengths for all three kits and tissues respectively by site.
Site 2 sequenced to a length of 76 nts, whereas all of Site1 samples were sequenced to <=50 nts.

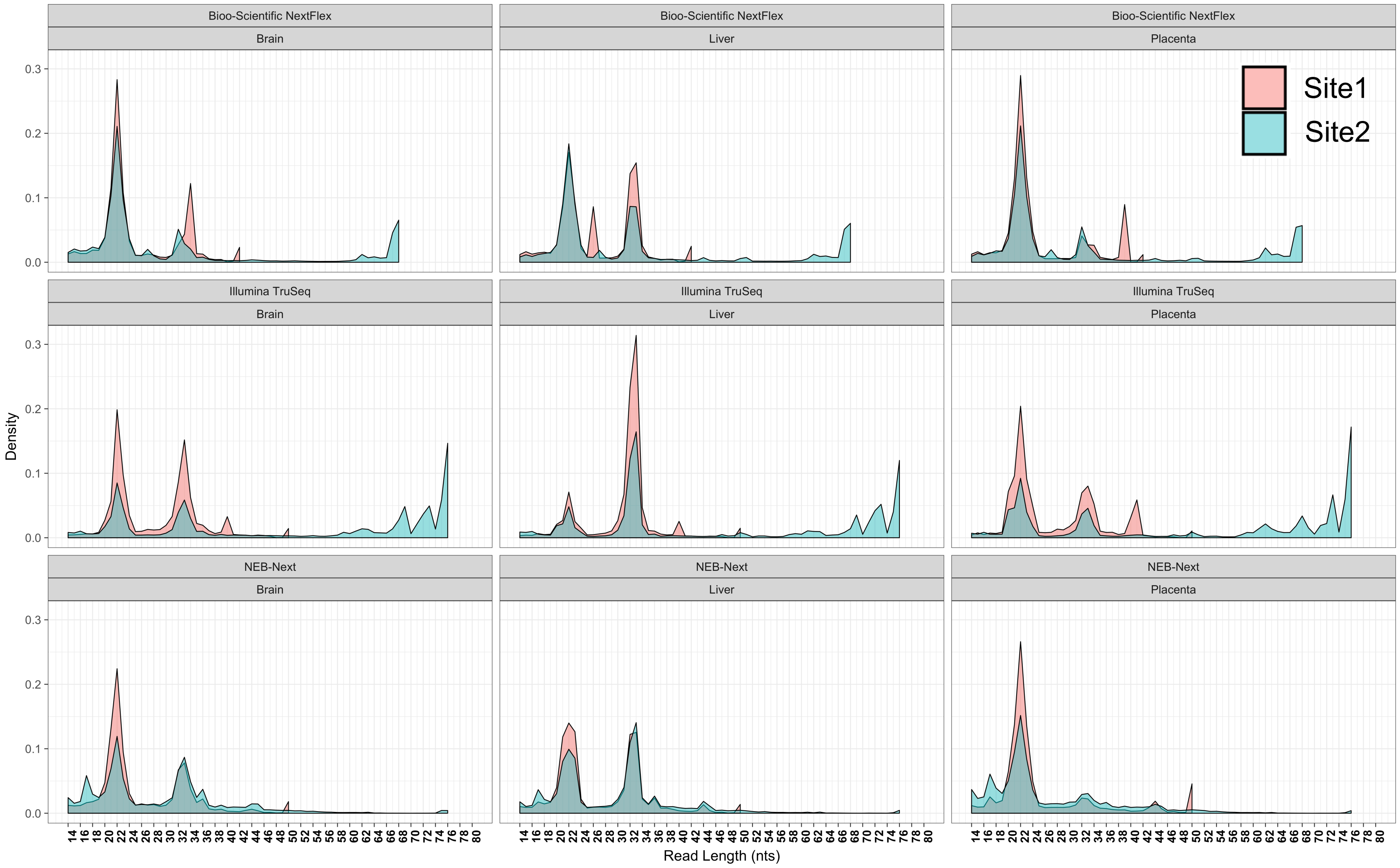

Figure S2 Comparison of percentage of reads assigned to the various RNA biotypes for read length restricted to less than 50 nts versus read length = 76 nts. Site2 sequenced to a length of 76 nts.

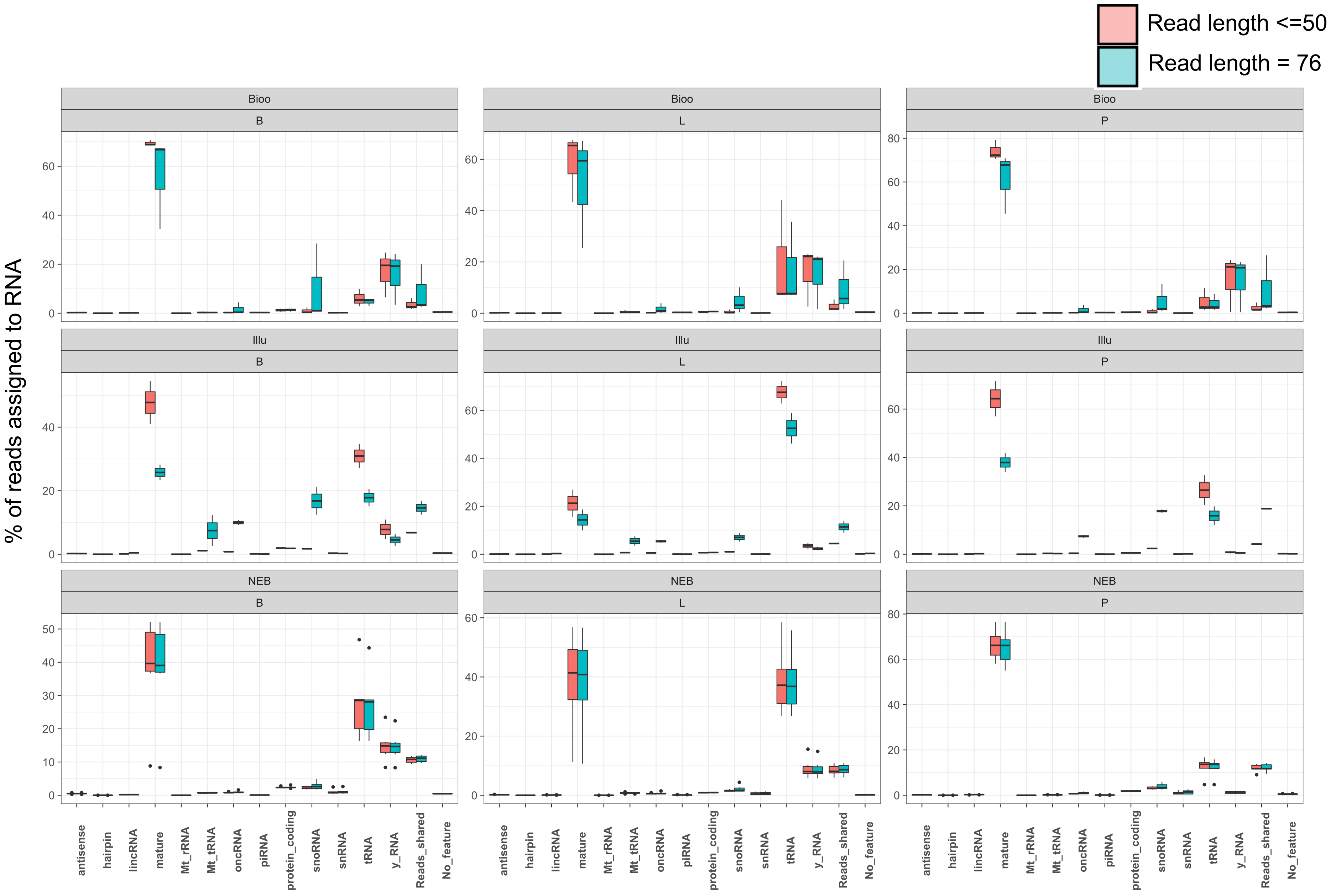

Figure S3. CA plot showing that the BiooScientificNextFlexsamples from Site2 cluster by themselves indicating a batch effect. Also, th

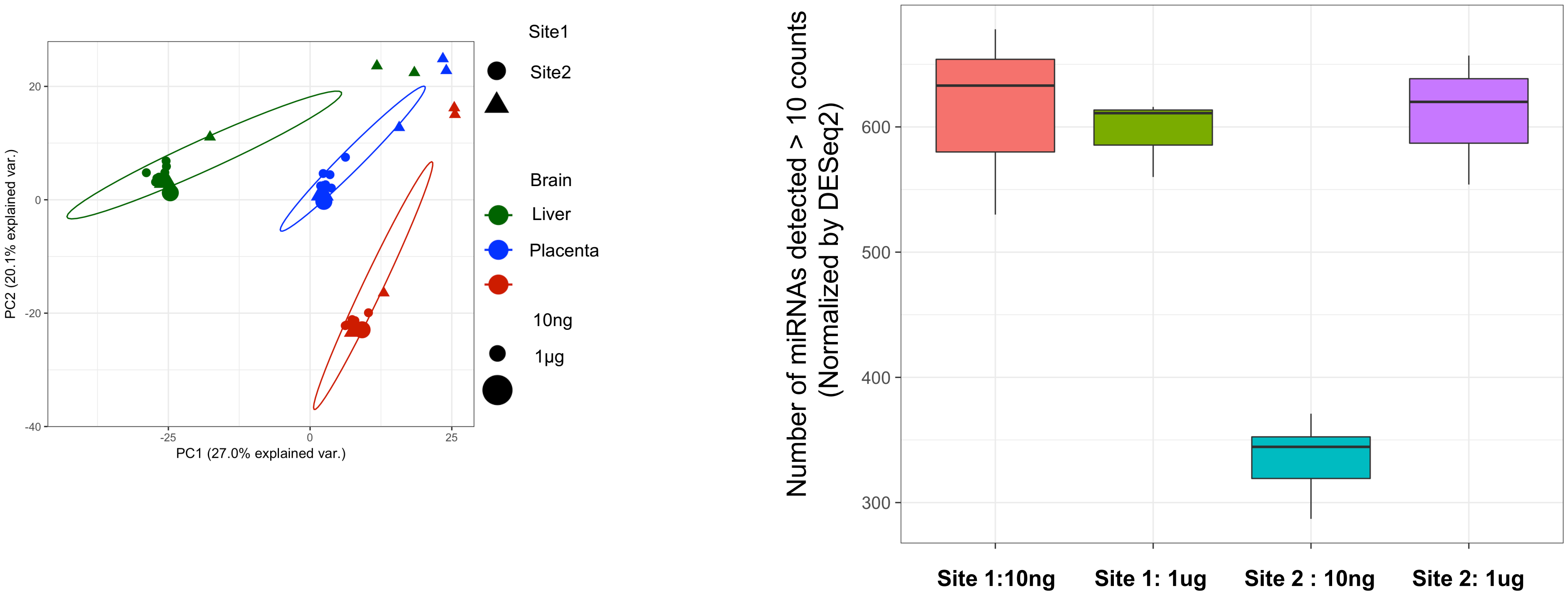

Supplement: Supplementary file 2 — Figure S1. Density plot of read lengths for all three kits and tissues respectively by site. Site2 sequenced to a length of 76 nts, whereas all of Site1 samples were sequenced to <=50 nts. Figure S2. Comparison of percentage of reads assigned to the various RNA biotypes for read length restricted to less than 50 nts versus read length = 76 nts. Site2 sequenced to a length of 76 nts. Figure S3. PCA plot showing that the BiooScientific NEXTFlex samples from Site2 cluster by themselves indicating a batch effect. Also, the figure on the right shows the number of miRNAs detected > 10 counts for the two input amounts 10 ng and 1 μg by Site for the BiooScientific NEXTFlex samples. (PDF 5418 kb) [file 12864_2018_4726_MOESM2_ESM.pdf]
